# Multi-Scale Spatio-Temporal Feature Extraction and Depth Estimation from Sequences by Ordinal Classification

**DOI:** 10.3390/s20071979

**Published:** 2020-04-01

**Authors:** Yang Liu

**Affiliations:** 1 School of Digital Media & Design Arts, Beijing University of Posts and Telecommunications, Beijing 100876, China; yang.liu@bupt.edu.cn; 2 Beijing Key Laboratory of Network System and Network Culture, Beijing 100876, China

**Keywords:** depth prediction, deep learning, spatio-temporal feature extraction, ConvLSTM, ordinal classification

## Abstract

Depth estimation is a key problem in 3D computer vision and has a wide variety of applications. In this paper we explore whether deep learning network can predict depth map accurately by learning multi-scale spatio-temporal features from sequences and recasting the depth estimation from a regression task to an ordinal classification task. We design an encoder-decoder network with several multi-scale strategies to improve its performance and extract spatio-temporal features with ConvLSTM. The results of our experiments show that the proposed method has an improvement of almost 10% in error metrics and up to 2% in accuracy metrics. The results also tell us that extracting spatio-temporal features can dramatically improve the performance in depth estimation task. We consider to extend this work to a self-supervised manner to get rid of the dependence on large-scale labeled data.

## 1. Introduction

Depth estimation [1,2,3,4,5,6,7] is a longstanding and fundamental task in 3D computer vision and enables a wide variety of applications, e.g., autonomous driving [8,9,10], Augmented Reality (AR) and Virtual Reality (VR) [11,12], Simultaneous Localization And Mapping (SLAM) [13,14,15,16,17,18], 2D-3D video conversion [19] and 3D scene understanding [20,21]. Most methods to estimate depth fall into three categories: Monocular Depth Estimation, Stereo Depth Estimation and Depth from Motion (Sequence).

Monocular Depth Estimation [22,23,24,25,26,27,28] infers depth information from single RGB images and is demonstrated to be an ill-posed problem(In most cases, there are several possible outputs corresponding to a given input image and the problem can be seen as a task of selecting the most proper one from all the possible outputs [29]). Stereo Depth Estimation [30,31,32] needs specific devices to capture stereo images and can provide much more clues to estimate the depth than Monocular Depth Estimation. Depth from Motion or Sequence [33,34,35,36] tries to predict the depth of each pixel by taking successive frames into account, which is the most common situation in our life and many applications. We find that most recently proposed methods focus on Monocular Depth Estimation, as it is more difficult to solve academically. However, such methods ignore one of the most important features for determining depth in the human vision system, which is motion, and in most applications, the format of input is in sequence.

In this paper, we concentrate on depth estimation from monocular sequences by using a single moving camera. This choice is motivated because monocular systems have higher efficiency compared with other approaches. Another reason is that the processing of this three-dimensional spatio-temporal signal is also a key problem in Signal Processing and Machine Learning [37,38].

Before the age of Deep Learning, most methods [13,16,39] extracted and matched local features [40,41] from RGB images between neighbouring frames and got the pose of camera and depth information following the theory of Multiple view of Geometry [42]. Recently, Deep Learning [43] has been gradually applied into this field. Reference [44] regards the depth estimation as a pixel-wise regression problem and designs a multi-scale coarse-to-fine deep neural network to handle this problem. References [25,45] design a network to estimate the pose and depth from sequences in an unsupervised manner, but they just take single RGB images as input when predicting the depth, which is used as the intermediate results in their loss functions. Reference [46] predicts depth from video and extracts spatio-temporal features by ConvLSTM.

Most existing methods fail to extract the spatio-temporal features embedded in the sequences or do not make better use of it [46]. Moreover, it is observed that the uncertainty in depth prediction increases along with the underlying ground-truth depth, which indicates that it would be better to allow a relatively larger error when predicting a larger depth value to avoid over-strengthened influence of large depth values on the training process [47].

In this paper, we design a deep neural network to estimate the depth from sequences and our contributions are as follows:

1. We design an encoder-decoder neural network with ConvLSTM to extract the spatio-temporal features from sequences.
2. We combine several multi-scale strategies [48,49] to achieve an accurate high resolution estimation.
3. Experimental results show that our network improves the performance of depth prediction.
4. Experimental results show that spatio-temporal features play an important role in depth prediction.

This paper is organized as follows: In Section 2, we briefly review some related work. In Section 3, our proposed method is introduced in details. The experimental results can be found in Section 4. Finally, Section 5 concludes this paper.

## 2. Related Work

### 2.1. ConvLSTM

Long Short-Term Memory (LSTM) [50] is one of the most famous building-blocks of Recurrent Neural Network (RNN). It is proved to be stable and powerful in modeling long-range dependencies for sequences.

**Figure 1:**
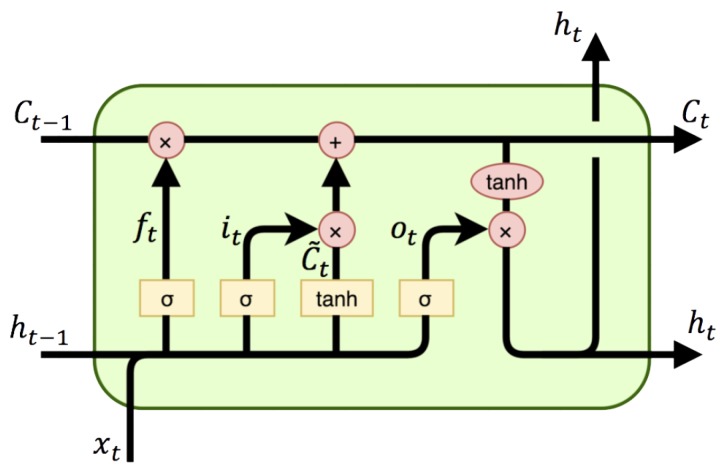
Structure of Long Short-Term Memory (LSTM).

LSTM, as shown in Figure 1, has a memory cell $C_{t}$ which essentially acts as an accumulator of the state information. The cell is accessed, written and cleared by several self-parameterized controlling gates. Every time a new input comes, its information will be accumulated to the cell if the input gate it is activated. In addition, the past cell status $C_{t-1}$ could be “forgotten” in this process if the forget gate $f_{t}$ is on. Whether the latest cell output $C_{t}$ will be propagated to the final state $h_{t}$ is further controlled by the output gate $o_{t}$. The key equations of LSTM are as below:

(1) $$\begin{array}{c} f_{t}=\sigma \left(W_{f}^{x}*x_{t}+W_{f}^{h}*h_{t-1}\right)\\ i_{t}=\sigma \left(W_{i}^{x}*x_{t}+W_{i}^{h}*h_{t-1}\right)\\ \tilde{C_{t}}=\tanh\left(W_{\tilde{C}}^{x}*x_{t}+W_{\tilde{C}}^{h}*h_{t-1}\right)\\ o_{t}=\sigma \left(W_{o}^{x}*x_{t}+W_{o}^{h}*h_{t-1}\right)\\ C_{t}=f_{t}\circ C_{t-1}+i_{t}\circ {\tilde{C}}_{t}\\ h_{t}=o_{t}\circ \tanh\left(C_{t}\right) \end{array}$$

where ∗ denotes the matrix multiplication.

The major drawback of traditional LSTM in handling spatio-temporal data is its usage of full connections in input-to-state and state-to-state transitions in which no spatial information is encoded [51]. ConvLSTM makes all the inputs $x_{1},\cdots ,x_{t}$, cell outputs $C_{1},\cdots ,C_{t}$, hidden states $h_{1},\cdots ,h_{t}$, and gates $i_{t},f_{t},o_{t}$ to be 3D tensors whose last two dimensions are spatial dimensions (rows and columns). So ConvLSTM can take RGB images or feature maps from convolution neural network as input. The key equations of ConvLSTM are the same in Equation (1), but the * denotes the convolution operator.

### 2.2. Ordinal Classification

When a variable is ordinal [52,53], its categories can be ranked from low to high, but the distances between adjacent categories are unknown. For example, if someone asks you about an idea, whether you strongly agree, agreed, have no opinion, disagree or strongly disagree with it, your opinion is ordinal.

**Figure 2:**
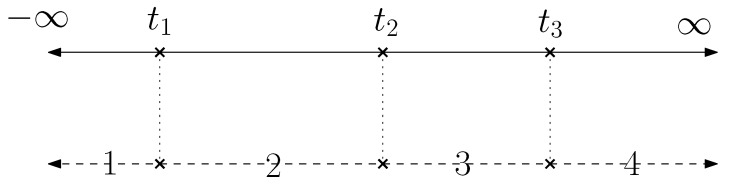
An example of ordinal classification.

Figure 2 shows a concrete example. We denote *x* as the features and β as learnable weights, ϵ is the random noise. The label *y* of this ordinal classification problem can be determined as follows.

$$\begin{array}{c} y=\left\{\begin{array}{cc} 1 & -\infty \leq \beta^{T}x+\epsilon <t_{1}\\ 2 & t_{1}\leq \beta^{T}x+\epsilon <t_{2}\\ 3 & t_{2}\leq \beta^{T}x+\epsilon <t_{3}\\ 4 & t_{3}\leq \beta^{T}x+\epsilon <\infty \end{array}\right. \end{array}$$

We can find that( $t_{0}=-\infty$ and $t_{4}=\infty$)

$$\begin{array}{cc} P(y=i|x) & =P\left(t_{i-1}\leq \beta^{T}x+\epsilon <t_{i}\right)=P\left(t_{i-1}-\beta^{T}x\leq \epsilon <t_{i}-\beta^{T}x\right)\\ \; & =F\left(t_{i}-\beta^{T}x\right)-F\left(t_{i-1}-\beta^{T}x\right) \end{array}$$

where F(·) is the cumulative density function of ϵ.

We can write the log-likelihood function:

$$\begin{array}{c} L\left(\beta \right)=\sum_{j}\sum_{k:y_{k}=j}\log\left[F\left(t_{j}-\beta^{T}x_{k}\right)-F\left(t_{j-1}-\beta^{T}x_{k}\right)\right] \end{array}$$

This expression can be maximized with numerical methods to estimate β.

## 3. Our Method

### 3.1. Motivation

We describe the motivations of this paper from three aspects.

#### 3.1.1. Depth from Sequences

We find that most applications [8,11,14] of depth estimation need to predict the depth of every frame from sequences. It is also known that successive frames in a sequence are highly related and embed numerous information of motion, which is believed to be important to predict depth [46]. So, it is a natural choice to train and test our model from sequences.

#### 3.1.2. ConvLSTM

There are three main methods to extract spatio-temporal features, CNN-RNN [54], 3D CNN [55], ConvLSTM [51]. CNN-RNN first extracts spatial features by a CNN network and then sends the results to an RNN network for temporal features. It extracts two kinds of features separately and works very well in some applications such as image captioning [54]. 3D CNN extract spatio-temporal features simultaneously, but it is hard to train [55]. ConvLSTM introduces convolution to traditional LSTM and takes images as input. Both spatial and temporal features are extracted in this unit.

#### 3.1.3. Ordinal Classification

As mentioned above, we are more confident about small depth, and should allow a relatively larger error when predicting a larger depth value. Most existing depth prediction deep learning networks output inverse depth or depth in log-space to solve this problem, and [47] shows that ordinal classification is another choice and achieves better performance. So we follow the idea in [47] and recast the depth estimation as an ordinal classification task.

### 3.2. Overview

**Figure 3:**
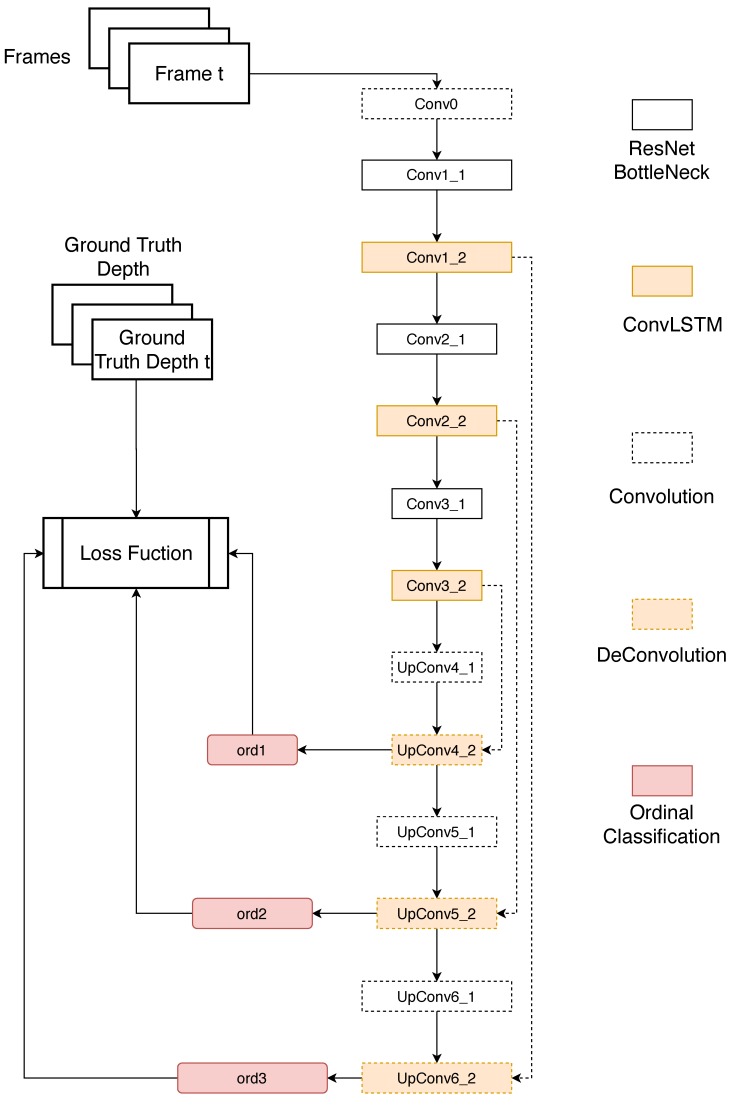
Architecture of the proposed network.

The architecture of our proposed network can be found in Figure 3. It is an encoder-decoder [56] architecture. The encoder part consists of three ResNet [57] Bottleneck layers and ConvLSTM layers, it then extracts the spatio-temporal features of input images. The decoder part restores the original resolution of images by three Convolution layers and DeConvolution [58] layers. The Ordinal classification layers are attached to the DeConvolution layer to recover the depth map.

The structure of our network can be found in Table 1. Conv(3 × 3 × 32) and ConvLSTM(3 × 3 × 32) means the kernel size is 3 × 3, the number of kernel is 32, the default value of stride is 1. DeConv(3 × 3 × 128.2) means the kernel size is 3 × 3, the number of kernel is 128, the stride is 2.

In the training stage, our network takes sequences(Every sequence is made up of three frames in our experiments) as input, ground-truth depth maps of corresponding frames as supervision. The encoder part tries to extract the embedded spatio-temporal features of input and obtains its feature map at 1/8 of original resolution (Conv3_2). Then the decoder attempts to recover the depth map from feature maps at different scale and compared the estimated depth map to the ground truth to obtain the error for back-propagation.

In the test stage, we just remove the Ordinal classification layers at ord1 and ord2 to estimate the depth map.

Details of our network are described in the following sections.

### 3.3. Multi-Scale Strategies

Pooling [43] is an important part of the deep convolution neural network. It reduces the complexity of the network and makes the model be invariant to some transformation. It is widely used in the task of classification [57,59], because classification needs to infer global information from the input, while depth estimation recovers local information from input. The repeated spatial pooling layers quickly reduce the spatial resolution of feature maps [47] (usually stride of 2), which is considered to have bad influence on performance of this task.

However, it is difficult to totally remove the pooling layers from the network. Hence, we adopt a multi-scale estimation to handle this problem.

#### 3.3.1. Location of Pooling Layer

It is difficult to remove all pooling layers in network practically, or the number of parameters of the network will explode. So we put them just after the ConvLSTM layers, as shown in Table 1. Firstly, ResNet Bottleneck extracts spatial features at the same resolution with its input and sends them to the following ConvLSTM layer. Then, we perform a max pooling on the feature map from ConvLSTM to decrease the spatial resolution of feature maps. Although the spatial resolution is decreased, the important local information is encoded, extracted and stored in the ConvLSTM ahead.

#### 3.3.2. Skip-Connection

In our network, there are three skip-connections [19] across the encoder and decoder to directly fuse the features at high resolution from the encoder to the decoder. This is one of the common strategies to improve the performance. Note that the width and height of input should be divisible by eight, or some error will occur in the decoder when concatenating them. For instance, if the size of input is 254 × 254 × 3, then the output size of Conv1_2, Conv2_2, Conv3_2 should be 127 × 127 × 64, 63 × 63 × 128, and 31 × 31 × 256. The output size of UpConv4_2 and UpConv5_1 is 62 × 62 × 128. Conv5_2 concatenates the output of Conv2_2 and UpConv5_1 and take them as input, so error occurs if their size is different.

#### 3.3.3. Multi-Scale Estimation

In the training stage, our model outputs three depth maps at 1/4 (ord1), 1/2 (ord2) and original (ord3) resolution with an input frame. They are compared to the corresponding resized ground truth depth to calculate the error. This multi-scale estimation [44] forces the decoder of our model to recover the depth map progressively, and fine-tune the estimation from low resolution to high resolution.

### 3.4. Spacing-Increasing Discretization

Most existing methods regard the depth estimation as a pixel-wise regression problem. However, few methods output the depth directly, because it is well-known that the uncertainty in depth prediction increases along with the underlying ground-truth depth, which indicates that it would be better to allow a relatively larger error when predicting a larger depth value to avoid over-strengthened influence of large depth values on the training process [47]. A common solution is performing the regression in log space, but the results are still unsatisfactory [47].

Another idea is recasting the depth estimation as an ordinal classification task, and quantify the continuous depth value to several discrete label values. However, when the depth value becomes larger and larger, its confidence reduces dramatically, which means that the estimation error of larger depth values is generally larger. Hence, using the uniform discretization strategy would induce an over-strengthened loss for the large depth values. We adopt a Spacing-Increasing Discretization proposed in [47], which uniformly discretizes a given depth interval in log space to down-weight the training losses in regions with large depth values, so that our depth estimation network is capable to more accurately predict relatively small and medium depth and to rationally estimate large depth values.

Assuming that a depth interval [a,b] needs to be discretized into *K* sub-intervals.

(2) $$\begin{array}{cc} \; & a=t_{0}=e^{s_{0}},s_{0}=\ln\left(a\right) \end{array}$$

(3) $$\begin{array}{cc} \; & b=t_{K}=e^{s_{K}},s_{K}=\ln\left(b\right) \end{array}$$

(4) $$\begin{array}{cc} \; & t_{i}=e^{s_{i}},i\in \left[0,K\right] \end{array}$$

The array ${\left\{s_{i}\right\}}_{0}^{K}$ is an arithmetic sequence, and

(5) $$\begin{array}{cc} \; & s_{i}=s_{0}+\frac{i}{K}\left(s_{K}-s_{0}\right)=\ln\left(a\right)+\frac{i}{K}\left(\ln\left(b\right)-\ln\left(b\right)\right) \end{array}$$

(6) $$\begin{array}{cc} \; & t_{i}=e^{s_{i}}=a{\left(\frac{b}{a}\right)}^{i/k} \end{array}$$

**Figure 4:**
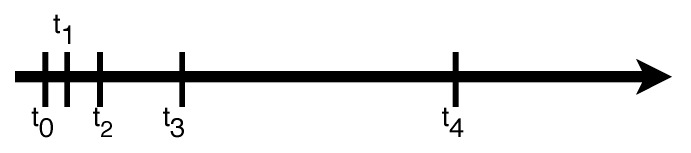
An example of Spacing-Increasing Discretization.

Figure 4 shows an example of Spacing-Increasing Discretization.

### 3.5. Ordinal Classification

After obtaining the discrete depth values, it is straightforward to turn the standard regression problem into an ordinal classification [47] problem.

Let $X_{i}=\phi \left(I\right)$ denote the output of DeConvolution layer at different scale (i=1 for UpConv4_2, i=2 for UpConv5_2, i=3 for UpConv6_2). $Y_{i}=\Psi \left(X_{i},\Theta_{i}\right)$ denotes the output of ordinal classification layer given $X_{i}$ with parameters $\Theta_{i}$ at scale *i* of size $W_{i}\times H_{i}\times 2K$. $W_{i}$ and $H_{i}$ are the width and height at scale *i*, and the value of depth are discretized into *K* sub-intervals. $Y_{i}\left[w,h,2j+1\right]>Y_{i}\left[w,h,2j\right]$ means the estimated ordinal labes ${\hat{l}}_{\left(w,h\right)}>j$. $\Theta_{i}=\left\{\theta_{0}^{i},\theta_{1}^{i},\cdots ,\theta_{2k-1}^{i}\right\}$ are the parameters. The loss function at *i*th scale can be formulated as follows.

(7) $$\begin{array}{cc} \; & L_{i}=-\frac{1}{N}\sum_{w}\sum_{h}\Lambda_{i}\left(w,h,X_{i},\Theta_{i}\right) \end{array}$$

(8) $$\begin{array}{cc} \; & \Lambda_{i}\left(w,h,X_{i},\Theta_{i}\right)=\sum_{k=0}^{l_{\left(w,h\right)}-1}\log\left(P_{\left(w,h\right)}^{i,k}\right)+\sum_{k=l_{\left(w,h\right)}}^{K-1}\log\left(1-P_{\left(w,h\right)}^{i,k}\right) \end{array}$$

(9) $$\begin{array}{cc} \; & P_{\left(w,h\right)}^{i,k}=P\left({\hat{l}}_{\left(w,h\right)}>k|X_{i},\Theta_{i}\right) \end{array}$$

where $l_{\left(w,h\right)}$ is the ground truth label of depth of image located at (w,h), ${\hat{l}}_{\left(w,h\right)}$ is the estimated label of depth if image located at (w,h). Equation (7) is the log-likelihood function at (w,h). $P_{\left(w,h\right)}^{i,k}$ is calculated as follows

(10) $$\begin{array}{c} P_{\left(w,h\right)}^{i,k}=\frac{e^{y_{\left(w,h,2k+1\right)}^{i}}}{e^{y_{\left(w,h,2k\right)}^{i}}+e^{y_{\left(w,h,2k+1\right)}^{i}}} \end{array}$$

where $y_{\left(w,h,2k+1\right)}^{i}={\left(\theta_{2k+1}^{i}\right)}^{T}x_{\left(w,h\right)}^{i}$, and $x_{\left(w,h\right)}^{i}\in X_{i}$.

In the inference phase, after obtaining ordinal labels for each pixel of image, the predicted depth value can be calculated as follow(We ignore the superscript i of $P_{\left(w,h\right)}^{i,k}$ for simplicity):

(11) $$\begin{array}{cc} {\hat{l}}_{\left(w,h\right)}= & \sum_{k=0}^{K}I\left(P_{\left(w,h\right)}^{k}\geq 0.5\right) \end{array}$$

(12) $$\begin{array}{cc} {\hat{d}}_{\left(w,h\right)}= & \frac{t_{{\hat{l}}_{\left(w,h\right)}}+t_{{\hat{l}}_{\left(w,h\right)}+1}}{2} \end{array}$$

where $t_{i}$ is the discretized depth value, as shown in Figure 4, I(·) is the indicator function, which means I(true)=1 and I(false)=0.

### 3.6. Loss Function

The final loss function of our model is the sum of loss functions at different scale with weight parameters.

(13) $$\begin{array}{c} L=\lambda_{1}L_{1}+\lambda_{2}L_{2}+\lambda_{3}L_{3} \end{array}$$

Parameters $\lambda_{1},\lambda_{2},\lambda_{3}$ balance the weights among three scales and we set $\lambda =1/4,\lambda_{2}=1/2,$$\lambda_{3}=1$ in the following experiments.

## 4. Experimental Results

Our model was trained and evaluated on the KITTI dataset [60]. The KITTI dataset consisted of video sequences of outdoor scenes along with their corresponding depth maps, procured using car-mounted cameras and Velodyne LiDAR sensors. We split the KITTI dataset into train and test following the description in [44], and we trained on 28 sequences and tested on the 697 images provided in [44]. The range of depth was set from 0 to 80 m, which meant a=0,b=80, and the depth was divided into K=70 sub-intervals. Throughout our experiments, the time-step of the ConvLSTM was set to 3. We evaluated our approach by using the standard metrics proposed by [44].

We compared the methods in two commonly used metrics: error metric and accurate metric. We denote *y* as the predicted depth and $y^{*}$ as ground truth depth, the key equations of these metrics are as follows:

$$\begin{array}{cc} \mathrm{Accuracy}\;\mathrm{Metric}:\; & \;\mathrm{percent}\;\mathrm{of}\;y\;\mathrm{that}\;\max(\frac{y}{y^{*}},\frac{y^{*}}{y})=\delta \\ \mathrm{Abs}\;\mathrm{Rel}:\; & \;\frac{1}{N}\sum \left|y-y^{*}\right|/y^{*}\\ \mathrm{Sq}\;\mathrm{Rel}:\; & \;\frac{1}{N}\sum {\left(y-y^{*}\right)}^{2}/y^{*}\\ \mathrm{RMSE}:\; & \;\sqrt{\frac{1}{N}\sum {\left(y-y^{*}\right)}^{2}}\\ \mathrm{RMSE}\;\mathrm{Log}:\; & \;\sqrt{\frac{1}{N}\sum {\left(\log\left(y\right)-\log{\left(y\right)}^{*}\right)}^{2}} \end{array}$$

We conducted three experiments on our proposed method. Our${}_{vo}$ means the model took sequences as input and got results from the ordinal classification layer as shown in Figure 3. Our${}_{vc}$ replaced ordinal classification layers with convolution layers, namely Conv(3 × 3 × 1), and they were fine-tuned with training data. We trained this model to investigate the importance of ordinal classification layer. Our${}_{so}$ means the model took single RGB images as input and got results from the ordinal classification layer. Although the network was trained using sequences, the decoder part was designed to individually recover each state of the phase of the encoder. Doing so allowed us to use a single image as input, estimate its depth map, and compare its results to Our${}_{vo}$ to find out the how much performance improved by taking sequences as input.

Table 2 shows the experimental results. We compared the proposed method with existing supervised method: DORN [47], DepthNet [46], Kuznietsov [22], and Eigen [44]. These four methods were trained with depth supervision, and Kuznietsov [22] had extra pose supervision.

In our experiments, we trained five different models for Our${}_{vo}$ and Our${}_{vc}$ respectively with the same training data and random initialization; the results of Our${}_{vo}$, Our${}_{vc}$ and Our${}_{so}$ are the average of 5 runs for each model.

The results show that Our${}_{vo}$ outperformed other methods. If compared with DORN, that had the best performances among four existing methods, Our${}_{vo}$ had a 13.88% improvement in Abs Rel (0.072->0.062), a 14% improvement in Sq Rel (0.307->0.264), a 15.18% improvement in RMSE (2.727->2.313), a 15.83% improvement in RMSE Log (0.307->0.264), a 2 percent point improvement when δ<1.25 (93.2->95.3%), a 0.7 percent point improvement when $\delta <1.25^{2}$ (98.4->99.1%), a 0.1 percent point improvement when $\delta <1.25^{3}$ (99.4->99.5%). We can also get some conclusion by comparing the performance of several groups of methods.

Firstly, Our${}_{vc}$ had better results than DepthNet. Both of them took sequences as input, utilized ConvLSTM to extract spatio-temporal features, got output from the convolution layer; they also had similar network architecture. Nevertheless, our${}_{vc}$ chose ResNet Bottleneck to extract features from images and adopted a multi-scale strategy, by which we believe improvement was brought.

Secondly, the performance of Our${}_{vo}$ was better than DORN, while the performance of DORN was better than Our${}_{so}$. The main difference between DORN and Our${}_{so}$ was the architecture of the network. DORN designed a complex network with dense feature extractor, multi-scale feature learner, cross channel information learner and a full-image encoder, while Our${}_{so}$ lost its ability to infer through the time for taking single RGB images as input. However, Our${}_{vo}$ beat DORN with a much simpler network which indicates the importance of temporal feature in depth estimation.

Finally, Our${}_{vc}$ achieved better results than Our${}_{so}$ which shows that temporal feature may play a much more important role than ordinal classification layer.

**Figure 5:**
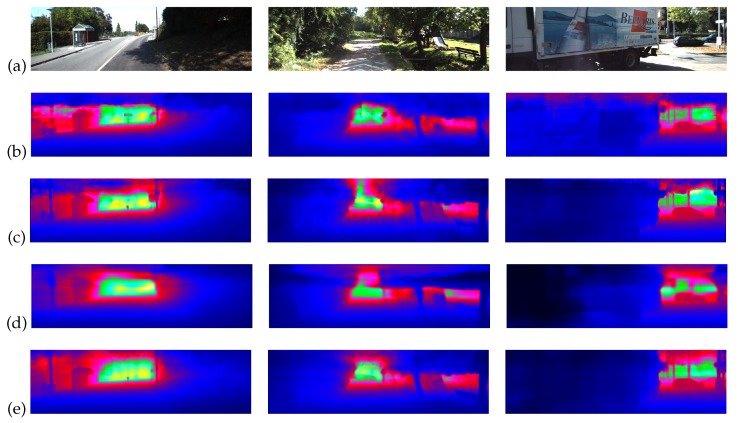
Visual results. (**a**) The first row is the input RGB images, (**b**) the second row is the results of DORN [47], (**c**) the third row is the results of Our${}_{so}$, (**d**) the fourth row is the results of Our${}_{vc}$, and (**e**) the last row is the results of Our${}_{vo}$

Some visual result can be found in Figure 5. The results of DORN and Our${}_{so}$ was sharper and more jittery than Our${}_{vc}$ and Our${}_{vo}$, because Our${}_{vc}$ and Our${}_{vo}$ took sequences as input and had the ability to smooth the output in the time domain.

## 5. Conclusions

In this paper, we design a deep learning neural network to extract spatio-temporal features from sequences, motivated by [46], and predict the depth map from it by ordinal classification, inspired by [47]. The network encodes the input by ResNet Bottleneck and ConvLSTM, then decodes and recovers the resolution of input images by Convolution and DeConvolution with skip-connection from encoder. We train the network with a multi-scale loss function to improve the performance. The results of our experiments show that the proposed method has an improvement of almost 10% in error metrics and up to 2% in accuracy metrics when comparing with the recently proposed supervised depth estimation methods. These results also show us that extracting temporal features can significantly improve the performance in depth estimation task. In the future, we will follow this work and design a self-supervised network to get rid of the dependence on large-scale labeled data.

## Figures and Tables

**Table 1:** Structure of the proposed network.

Layers	Structure	Input Size	Output Size
Conv_0	Conv(3 × 3 × 32)	H × W × 3	H × W × 32
	Conv(1 × 1 × 16)	H × W × 32	H × W × 16
Conv1_1	Conv(3 × 3 × 16)	H × W × 16	H × W × 16
	Conv(1 × 1 × 32)	H × W × 16	H × W × 32
Conv1_2	ConvLSTM(3 × 3 × 64)	H × W × 32	H × W × 64
	MaxPooling	H × W × 64	H/2 × W/2 × 64
	Conv(1 × 1 × 32)	H/2 × W/2 × 64	H/2 × W/2 × 32
Conv2_1	Conv(3 × 3 × 32)	H/2 × W/2 × 32	H/2 × W/2 × 32
	Conv(1 × 1 × 64)	H/2 × W/2 × 32	H/2 × W/2 × 64
Conv2_2	ConvLSTM(3 × 3 × 128)	H/2 × W/2 × 64	H/2 × W/2 × 128
	MaxPooling	H/2 × W/2 × 128	H/4 × W/4 × 128
	Conv(1 × 1 × 64)	H/4 × W/4 × 128	H/4 × W/4 × 64
Conv3_1	Conv(3 × 3 × 64)	H/4 × W/4 × 64	H/4 × W/4 × 64
	Conv(1 × 1 × 128)	H/4 × W/4 × 64	H/4 × W/4 × 128
Conv3_2	ConvLSTM(3 × 3 × 256)	H/4 × W/4 × 128	H/4 × W/4 × 256
	MaxPooling	H/4 × W/4 × 256	H/8 × W/8 × 256
UpConv4_1	Conv(3 × 3 × 256)	H/8 × W/8 × 256	H/8 × W/8 × 256
UpConv4_2	DeConv(3 × 3 × 128.2)	H/8 × W/8 × 256	H/4 × W/4 × 128
UpConv5_1	Conv(3 × 3 × 128)	H/4 × W/4 × 128	H/4 × W/4 × 128
UpConv5_2	DeConv(3 × 3 × 64.2)	H/4 × W/4 × 128	H/2 × W/2 × 64
UpConv6_1	Conv(3 × 3 × 64)	H/2 × W/2 × 64	H/2 × W/2 × 64
UpConv6_2	DeConv(3 × 3 × 32.2)	H/2 × W/2 × 64	H × W × 32

**Table 2:** Results on KITTI.

Method	Error Metrics				Accuracy Metrics		
	Abs Rel	Sq Rel	RMSE	RMSE Log	δ<1.25	$\delta <1.25^{2}$	$\delta <1.25^{3}$
Eigen [44]	0.214	1.605	6.563	0.292	0.673	0.884	0.957
DepthNet [46]	0.137	1.019	5,187	0.218	0.809	0.928	0.971
Kuznietsov [22]	0.113	0.741	4.621	0.189	0.875	0.964	0.988
DORN [47]	0.072	0.307	2.727	0.120	0.932	0.984	0.994
Our${}_{so}$	0.129	0.865	4.663	0.194	0.881	0.959	0.972
Our${}_{vc}$	0.093	0.543	3.014	0.145	0.908	0.969	0.984
Our${}_{vo}$	**0.062**	**0.264**	**2.313**	**0.101**	**0.953**	**0.991**	**0.995**

## References

[1] Ha H., Im S., Park J., Jeon H.G., So Kweon I. High-quality depth from uncalibrated small motion clip. Proceedings of the IEEE Conference on Computer Vision and Pattern Recognition.

[2] Karsch K., Liu C., Kang S.B. (2014). Depth transfer: Depth extraction from video using non-parametric sampling. IEEE Trans. Pattern Anal. Mach. Intell..

[3] Kong N., Black M.J. Intrinsic depth: Improving depth transfer with intrinsic images. Proceedings of the IEEE International Conference on Computer Vision.

[4] Chen S., Tang M., Kan J. (2019). Predicting depth from single RGB images with pyramidal three-streamed networks. Sensors.

[5] Bhoi A. (2019). Monocular depth estimation: A survey. arXiv.

[6] Ummenhofer B., Zhou H., Uhrig J., Mayer N., Ilg E., Dosovitskiy A., Brox T. Demon: Depth and motion network for learning monocular stereo. Proceedings of the IEEE Conference on Computer Vision and Pattern Recognition.

[7] Kim S., Nam J., Ko B. (2019). Fast Depth Estimation in a Single Image Using Lightweight Efficient Neural Network. Sensors.

[8] Ding L., Feng C. DeepMapping: Unsupervised map estimation from multiple point clouds. Proceedings of the IEEE Conference on Computer Vision and Pattern Recognition.

[9] Zhang P., Ouyang W., Zhang P., Xue J., Zheng N. Sr-lstm: State refinement for lstm towards pedestrian trajectory prediction. Proceedings of the IEEE Conference on Computer Vision and Pattern Recognition.

[10] Palafox P.R., Betz J., Nobis F., Riedl K., Lienkamp M. (2019). SemanticDepth: Fusing Semantic Segmentation and Monocular Depth Estimation for Enabling Autonomous Driving in Roads without Lane Lines. Sensors.

[11] Alhaija H.A., Mustikovela S.K., Mescheder L., Geiger A., Rother C. (2018). Augmented reality meets computer vision: Efficient data generation for urban driving scenes. Int. J. Comput. Vision.

[12] Frikha R., Ejbali R., Zaied M. (2017). Camera pose estimation for augmented reality in a small indoor dynamic scene. J. Electron. Imaging.

[13] Cadena C., Carlone L., Carrillo H., Latif Y., Scaramuzza D., Neira J., Reid I., Leonard J.J. (2016). Past, present, and future of simultaneous localization and mapping: Toward the robust-perception age. IEEE Trans. Rob..

[14] Schonberger J.L., Frahm J.M. Structure-from-motion revisited. Proceedings of the IEEE Conference on Computer Vision and Pattern Recognition.

[15] Luo Z., Shen T., Zhou L., Zhu S., Zhang R., Yao Y., Fang T., Quan L. Geodesc: Learning local descriptors by integrating geometry constraints. Proceedings of the European Conference on Computer Vision (ECCV).

[16] Mur-Artal R., Tardós J.D. (2017). Orb-slam2: An open-source slam system for monocular, stereo, and rgb-d cameras. IEEE Trans. Rob..

[17] Liu W., Wu S., Wu Z., Wu X. (2019). Incremental Pose Map Optimization for Monocular Vision SLAM Based on Similarity Transformation. Sensors.

[18] Veiga Almagro C., Di Castro M., Lunghi G., Marín Prades R., Sanz Valero P.J., Pérez M.F., Masi A. (2019). Monocular Robust Depth Estimation Vision System for Robotic Tasks Interventions in Metallic Targets. Sensors.

[19] Xie J., Girshick R., Farhadi A. Deep3d: Fully automatic 2d-to-3d video conversion with deep convolutional neural networks. Proceedings of the European Conference on Computer Vision. Amsterdam.

[20] Zhang Y., Bai M., Kohli P., Izadi S., Xiao J. Deepcontext: Context-encoding neural pathways for 3d holistic scene understanding. Proceedings of the IEEE International Conference on Computer Vision.

[21] Zhuo W., Salzmann M., He X., Liu M. Indoor scene structure analysis for single image depth estimation. Proceedings of the IEEE Conference on Computer Vision and Pattern recognition.

[22] Kuznietsov Y., Stuckler J., Leibe B. Semi-supervised deep learning for monocular depth map prediction. Proceedings of the IEEE Conference on Computer Vision and Pattern recognition.

[23] Liu F., Shen C., Lin G., Reid I. (2015). Learning depth from single monocular images using deep convolutional neural fields. IEEE Trans. Pattern Anal. Mach. Intell..

[24] Roy A., Todorovic S. Monocular depth estimation using neural regression forest. Proceedings of the IEEE conference on computer vision and pattern recognition.

[25] Godard C., Mac Aodha O., Firman M., Brostow G.J. Digging into self-supervised monocular depth estimation. Proceedings of the IEEE International Conference on Computer Vision.

[26] Yin Z., Shi J. Geonet: Unsupervised learning of dense depth, optical flow and camera pose. Proceedings of the IEEE Conference on Computer Vision and Pattern Recognition, Salt Lake City.

[27] Mun J.H., Jeon M., Lee B.G. (2019). Unsupervised Learning for Depth, Ego-Motion, and Optical Flow Estimation Using Coupled Consistency Conditions. Sensors.

[28] Lin X., Sánchez-Escobedo D., Casas J.R., Pardàs M. (2019). Depth estimation and semantic segmentation from a single RGB image using a hybrid convolutional neural network. Sensors.

[29] Yoo J., Lee S.H., Kwak N. Image restoration by estimating frequency distribution of local patches. Proceedings of the IEEE Conference on Computer Vision and Pattern Recognition.

[30] Saxena A., Schulte J., Ng A.Y. Depth Estimation Using Monocular and Stereo Cues. Proceedings of the International Joint Conference on Artificial Intelligence (IJCAI).

[31] Smolyanskiy N., Kamenev A., Birchfield S. On the importance of stereo for accurate depth estimation: An efficient semi-supervised deep neural network approach. Proceedings of the IEEE Conference on Computer Vision and Pattern Recognition Workshops, Salt Lake City.

[32] Wang Y., Wang P., Yang Z., Luo C., Yang Y., Xu W. Unos: Unified unsupervised optical-flow and stereo-depth estimation by watching videos. Proceedings of the IEEE Conference on Computer Vision and Pattern Recognition.

[33] Valentin J., Kowdle A., Barron J.T., Wadhwa N., Dzitsiuk M., Schoenberg M., Verma V., Csaszar A., Turner E., Dryanovski I. (2018). Depth from motion for smartphone AR. ACM Trans. Graph..

[34] Aguilar-González A., Arias-Estrada M., Berry F. (2019). Depth from a motion algorithm and a hardware architecture for smart cameras. Sensors.

[35] Zhou J., Wang Y., Qin K., Zeng W. Unsupervised High-Resolution Depth Learning From Videos With Dual Networks. Proceedings of the IEEE International Conference on Computer Vision.

[36] Gordon A., Li H., Jonschkowski R., Angelova A. Depth from videos in the wild: Unsupervised monocular depth learning from unknown cameras. Proceedings of the IEEE International Conference on Computer Vision.

[37] Hu Y., Lu X. (2018). Learning spatial-temporal features for video copy detection by the combination of CNN and RNN. J. Visual Commun. Image Represent..

[38] Tang Q., Yang M., Yang Y. (2019). ST-LSTM: A deep learning approach combined spatio-temporal features for short-term forecast in rail transit. J. Adv. Transp..

[39] Klein G., Murray D. Parallel tracking and mapping for small AR workspaces. Proceedings of the 2007 6th IEEE and ACM International Symposium on Mixed and Augmented Reality.

[40] Lowe D.G. (2004). Distinctive image features from scale-invariant keypoints. Int. J. Comput. Vis..

[41] Rublee E., Rabaud V., Konolige K., Bradski G. ORB: An efficient alternative to SIFT or SURF. Proceedings of the 2011 International Conference on Computer Vision.

[42] Hartley R., Zisserman A. (2003). Multiple View Geometry in Computer Vision.

[43] LeCun Y., Bengio Y., Hinton G. (2015). Deep learning. Nature.

[44] Eigen D., Puhrsch C., Fergus R. Depth Map Prediction from a Single Image Using a Multi-Scale Deep network. http://papers.nips.cc/paper/5539-depth-map-prediction-from-a-single-image-using-a-multi-scale-deep-network.pdf.

[45] Zhou T., Brown M., Snavely N., Lowe D.G. Unsupervised learning of depth and ego-motion from video. Proceedings of the IEEE Conference on Computer Vision and Pattern Recognition.

[46] CS Kumar A., Bhandarkar S.M., Prasad M. Depthnet: A recurrent neural network architecture for monocular depth prediction. Proceedings of the IEEE Conference on Computer Vision and Pattern Recognition Workshops, Salt Lake City.

[47] Fu H., Gong M., Wang C., Batmanghelich K., Tao D. Deep ordinal regression network for monocular depth estimation. Proceedings of the IEEE Conference on Computer Vision and Pattern Recognition, Salt Lake City.

[48] Yu F., Koltun V. (2015). Multi-scale context aggregation by dilated convolutions. arXiv.

[49] Xu D., Ricci E., Ouyang W., Wang X., Sebe N. Multi-scale continuous crfs as sequential deep networks for monocular depth estimation. Proceedings of the IEEE Conference on Computer Vision and Pattern Recognition.

[50] Gers F.A., Schmidhuber J., Cummins F. Learning to forget: Continual prediction with LSTM. Proceedings of the 1999 Ninth International Conference on Artificial Neural Networks (ICANN 99).

[51] Xingjian S., Chen Z., Wang H., Yeung D.Y., Wong W.K., Woo W.C. Convolutional LSTM Network: A Machine Learning Approach for Precipitation Nowcasting. http://papers.nips.cc/paper/5955-convolutional-lstm-network-a-machine-learning-approach-for-precipitation-nowcasting.pdf.

[52] Frank E., Hall M. A simple approach to ordinal classification. Proceedings of the European Conference on Machine Learning.

[53] Zoran D., Isola P., Krishnan D., Freeman W.T. Learning ordinal relationships for mid-level vision. Proceedings of the IEEE International Conference on Computer Vision.

[54] Vinyals O., Toshev A., Bengio S., Erhan D. Show and tell: A neural image caption generator. Proceedings of the IEEE conference on computer vision and pattern recognition.

[55] Hara K., Kataoka H., Satoh Y. Can spatiotemporal 3d cnns retrace the history of 2d cnns and imagenet?. Proceedings of the IEEE Conference on Computer Vision and Pattern Recognition, Salt Lake City.

[56] Badrinarayanan V., Kendall A., Cipolla R. (2017). Segnet: A deep convolutional encoder-decoder architecture for image segmentation. IEEE Trans. Pattern Anal. Mach. Intell..

[57] He K., Zhang X., Ren S., Sun J. Deep residual learning for image recognition. Proceedings of the IEEE Conference on Computer Vision and Pattern Recognition.

[58] Noh H., Hong S., Han B. Learning deconvolution network for semantic segmentation. Proceedings of the IEEE International Conference on Computer Vision.

[59] Simonyan K., Zisserman A. (2014). Very deep convolutional networks for large-scale image recognition. arXiv.

[60] Geiger A., Lenz P., Stiller C., Urtasun R. (2013). Vision meets robotics: The kitti dataset. Int. J. Rob. Res..

